# Does pregnancy alter life-course lipid trajectories? Evidence from the HUNT Study in Norway[S]

**DOI:** 10.1194/jlr.P085720

**Published:** 2018-10-12

**Authors:** Amanda R. Markovitz, Eirin B. Haug, Julie Horn, Abigail Fraser, Corrie Macdonald-Wallis, Kate Tilling, Eric B. Rimm, Stacey A. Missmer, Paige L. Williams, Pål R. Romundstad, Bjørn O. Åsvold, Janet W. Rich-Edwards

**Affiliations:** Department of Epidemiology* Harvard T.H. Chan School of Public Health, Boston, MA; Department of Nutrition,§§ Harvard T.H. Chan School of Public Health, Boston, MA; Connors Center for Women’s Health and Gender Biology† Brigham and Women’s Hospital and Harvard Medical School, Boston, MA; Channing Division of Network Medicine,*** Department of Medicine, Brigham and Women’s Hospital and Harvard Medical School, Boston, MA; K.G. Jebsen Center for Genetic Epidemiology,**** Norwegian University of Science and Technology, Trondheim, Norway; Department of Public Health and Nursing,§ Norwegian University of Science and Technology, Trondheim, Norway; Department of Obstetrics and Gynecology,** Levanger Hospital, Nord-Trøndelag Hospital Trust, Levanger, Norway; Population Health Sciences,†† Bristol Medical School and MRC Integrative Epidemiology Unit at the University of Bristol, Bristol, UK; Division of Adolescent and Young Adult Medicine,††† Department of Pediatrics, Boston Children’s Hospital and Harvard Medical School, Boston, MA; Department of Obstetrics, Gynecology, and Reproductive Biology,§§§ College of Human Medicine, Michigan State University, Grand Rapids, MI; Department of Endocrinology,†††† St. Olavs Hospital, Trondheim University Hospital, Trondheim, Norway

**Keywords:** parity, cholesterol, high density lipoprotein, triglycerides, epidemiology, spline, mixed model, Nord-Trøndelag Health Study

## Abstract

We examined the association between pregnancy and life-course lipid trajectories. Linked data from the Nord-Trøndelag Health Study and the Medical Birth Registry of Norway yielded 19,987 parous and 1,625 nulliparous women. Using mixed-effects spline models, we estimated differences in nonfasting lipid levels from before to after first birth in parous women and between parous and nulliparous women. HDL cholesterol (HDL-C) dropped by −4.2 mg/dl (95% CI: −5.0, −3.3) from before to after first birth in adjusted models, a 7% change, and the total cholesterol (TC) to HDL-C ratio increased by 0.18 (95% CI: 0.11, 0.25), with no change in non-HDL-C or triglycerides. Changes in HDL-C and the TC/HDL-C ratio associated with pregnancy persisted for decades, leading to altered life-course lipid trajectories. For example, parous women had a lower HDL-C than nulliparous women at the age of 50 years (−1.4 mg/dl; 95% CI: −2.3, −0.4). Adverse changes in lipids were greatest after first birth, with small changes after subsequent births, and were larger in women who did not breastfeed. Findings suggest that pregnancy is associated with long-lasting adverse changes in HDL-C, potentially setting parous women on a more atherogenic trajectory than prior to pregnancy.

Lipids predict future CVD (1). Although recent evidence suggests that altering HDL cholesterol (HDL-C) levels does not itself cause changes in CVD risk (2), lower HDL-C may serve as a marker of a more atherogenic lipid profile. Pregnancy induces profound metabolic, endocrine, and cardiovascular changes that may have long-lasting or permanent effects in mothers. In the first 10 years after giving birth, changes in lipid levels have been observed (3–5). Although total cholesterol (TC) appears to return to prepregnancy levels within a year (3, 6–8), there is consistent evidence that HDL-C decreases postpartum and remains lower than prepregnancy levels for multiple years (3, 6–10) and less consistent evidence that triglycerides remain elevated postpartum (3, 6, 7, 9). However, no study has examined lipid trajectories beyond a decade after pregnancy (3, 6–8, 10). If adverse changes in lipids continue into midlife and beyond, it could provide insight into the early origins of subclinical CVD risk in women. In addition, few previous studies were able to characterize how lipid levels changed with time since pregnancy. Furthermore, few studies have considered breastfeeding as part of the peripartum year. Lactation is a modifiable factor that might minimize adverse changes in lipids postpartum and is associated with higher HDL-C levels (11–15) and a more rapid return of triglycerides to prepregnancy levels (16). A previous study found that women who breastfed longer had smaller postpartum decreases in HDL-C (17).

The population-based Nord-Trøndelag Health Study (HUNT), linked with the Medical Birth Registry of Norway, includes data on pregnancy, breastfeeding, and measured lipid values in women from before and up to 41 years after first birth, enabling an examination of changes in lipid levels pre- to postpregnancy. These data enable us to examine, for the first time, the impact of pregnancy on the ratio of TC to HDL-C, which performs as well as or better than other lipid measures in CVD risk prediction (18–24). Using these data, we investigated the association of first birth with short- and long-term changes in lipid levels. We also examined the impact of later births on lipid levels. Finally, we investigated the extent to which these changes differed by breastfeeding length.

## MATERIALS AND METHODS

### Study population

HUNT is a population-based open cohort study of adult Nord-Trøndelag county residents designed for a wide range of health-related research. County-wide surveys are conducted roughly every decade, with three completed at the time of this analysis. This analysis was restricted to the second and third surveys in which lipids were sampled: HUNT2 (1995–1997) and HUNT3 (2006–2008). During the surveys, participants received an extensive health assessment that included blood sampling, clinical measurements, and questionnaires (25). All current county residents aged 20 years or older identified from the national population register were invited to participate in each survey, with participation rates among women of 76% in HUNT2 (26) and 59% in HUNT3 (25). Residents of Nord-Trøndelag county are predominantly white and generally representative of Norway as a whole (26).

**Fig. 1.**
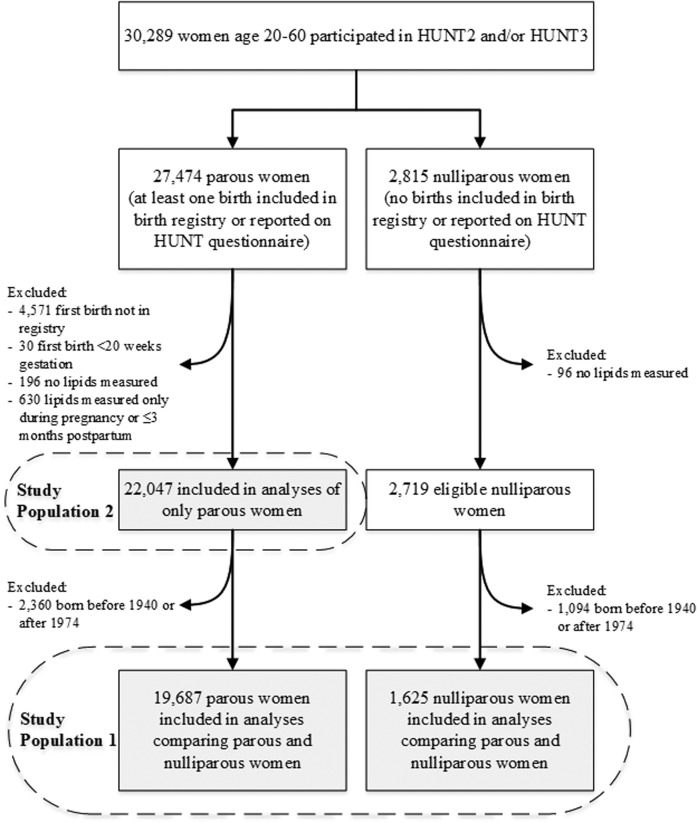
Flow chart of the study population.

We linked HUNT data to the Medical Birth Registry of Norway, which includes all births in Norway from 1967 (27) through the end of our data collection in 2012. Because older women were unlikely to have their pregnancy history captured in this registry, we restricted analyses to women aged 20–60 years during lipid measurement. **Figure 1** outlines the process of identifying two overlapping study populations. The first included parous and nulliparous women with similar age distributions to compare lipids trajectories between the two groups. The second included parous women to compare lipids before and after first birth. For the first population, we excluded women born before 1940 or after 1974 to prevent misclassification of women as nulliparous who had a birth before the birth registry started in 1967 or after the end of data collection in 2012. We applied this exclusion to parous women to achieve a comparable age distribution. For the second population of only parous women, we did not restrict based on birth year. All participants in HUNT signed an informed consent form allowing the use of their data and samples for research. This project was approved by the Central Norway Regional Committee for Medical and Health Research Ethics and was considered exempt from institutional review board review by the Harvard T. H. Chan School of Public Health. This study abides by the Declaration of Helsinki principles.

### Lipid and covariate assessment

Participants’ ages ranged from 20 to 60 years during measurements. Among parous women, 2,488 women had at least 1 measurement before their first birth (with a total of 2,521 prepregnancy observations), including 747 women with measurements both before and after first birth (supplemental Fig. S1). Nonfasting lipids were measured from serum samples. For technical details about TC, HDL-C, and triglyceride measurements, see supplemental Table S1. We calculated non-HDL-C as TC minus HDL-C and the TC/HDL-C ratio. LDL cholesterol (LDL-C) was not analyzed because the Friedewald formula (28), typically used to calculate LDL-C, performs poorly in nonfasting samples (29–31). At the time of lipid measurements, staff recorded the time since last meal in hour categories.

Covariates were collected during HUNT surveys and were selected based on the causal diagram shown in supplemental Fig. S2. We used data from all HUNT questionnaires, including HUNT1 (1984–1986), to identify the following time-invariant covariates: *1*) family history of CVD (any reported myocardial infarction or angina pectoris in siblings or parents); *2*) smoking status at the age of 20 years, defined as ever versus never smoked daily prior to or at the age of 20 years, to approximate prepregnancy smoking behavior for parous women; and *3*) highest obtained education level. HUNT3 did not collect education level and was instead derived from work titles for 13% of women based on recommendations from Statistics Norway (32). We also included the following time-varying, or updated, covariates: *1*) BMI, *2*) smoking status, *3*) alcohol use, *4*) vigorous leisure-time physical activity, and *5*) oral contraceptive use. Time-varying covariates were measured at the time of lipid assessment either from HUNT2 and HUNT3 clinical examinations (in the case of BMI) or HUNT2 and HUNT3 questionnaires (for all other covariates). We obtained additional information about first births, including maternal age and preterm status (<37 weeks gestation), from the birth registry and breastfeeding length after first birth (self-reported as 0, <3, 3–6, and >6 months) from HUNT questionnaires. We additionally obtained self-reported information about menopause transition and hormone replacement therapy (never, previous, or current user) from HUNT questionnaires.

### Analysis

We used linear mixed-effects models to estimate lipid trajectories as a function of age, accounting for the timing of a woman’s first birth. Age was modeled using restricted cubic splines with four knots located at ages 23, 37, 46, and 57 years based on prespecified quantiles of the age distribution, as recommended by Harrell (33). Two variables were used to estimate the effect of pregnancy. The first indicated whether measurement preceded or followed the first birth, providing an estimate of short-term change in lipids after first birth. The second indicated continuous time since first birth, providing an estimate of longer-term change in lipids postpartum. All models controlled for the participant’s age at measurement, HUNT survey (HUNT2 vs. HUNT3), time since last meal, education, smoking initiation by the age of 20 years, and family history of CVD.

First, we compared life-course lipid trajectories for parous and nulliparous women based on completed reproductive history. In this analysis (see supplemental Methods section 1), nulliparous women represented background age and secular trends independent of parity. These analyses included an indicator of final parity status (i.e., none vs. one or more births; covariate *P_i_* in supplemental Methods equation 1) and an interaction between parity and the spline terms, allowing the age-related splines to differ throughout the life course based on final parity status. We chose final rather than updated parity status because nulliparous and parous women are likely to be different even before the latter give birth, given observed associations between infertility and lipid levels (34, 35). For these and other analyses controlling only for baseline covariates, we used a complete case analysis, excluding participants with missing data on education (0.6%) or smoking (2.5%). To present the trajectories graphically, predicted lipid trajectories were derived for hypothetical nulliparous women and parous women with a first birth occurring at the age of 23 years (the median age at first birth in the study population), setting all other covariates to their mean levels.

Second, we used the same mixed-effects spline models among parous women (study population 2) to obtain estimates of the short-term effect of the first birth on lipid levels and to describe differences in this effect estimate by length of breastfeeding (supplemental Methods section 2). The short-term effect of the first birth was estimated based on the discontinuity between predicted trajectories before first birth and predicted trajectories after first birth (captured by the coefficient *I_ij_* in supplemental Methods equation 2). Fully adjusted results from these models included updated BMI, alcohol use, physical activity, and oral contraceptive use as well as maternal age and preterm status of first birth. Both baseline and updated covariates were multiply imputed using fully conditional specification (36) with 25 iterations. Models investigating whether breastfeeding length moderated the change in lipid levels from before to after first birth included distinct indicator terms for the postpartum versus prepartum effect based on breastfeeding length. Approximately 99% of lipid measurements informing after first birth trends in our analysis occurred after breastfeeding ended; thus, the before versus after first birth effect we estimated would include changes associated with breastfeeding. We used F-tests to determine whether the postpartum versus prepartum effect differed by breastfeeding length (37). Finally, we investigated the change in lipids after second and third births among women with multiple births.

### Sensitivity analyses

We performed sensitivity analyses among women with measurements at both HUNT2 and HUNT3 (47% of women) to verify that our main results, which included some women with only one measurement, were consistent with within-woman changes in lipids observed among women with more than one lipid measurement. First, we replicated the lipid trajectory models among women with repeated measures to verify that our results could be interpreted as within-woman life-course trajectories. Second, we compared the within-woman change in lipid levels from HUNT2 to HUNT3 for women who had one or more births during the ∼11 year interval to the within-woman lipid changes for women who remained nulliparous during the interval using a difference-in-differences approach (38). We additionally performed sensitivity analyses controlling for menopause transition and hormone replacement therapy to see whether this differentially affected life-course lipid trajectories for parous compared with nulliparous women. All analyses were performed using Stata IC 13 (StataCorp, College Station, TX) and MLwiN (39) version 2.34.

## RESULTS

Among 21,312 study participants born between 1940 and 1974 (study population 1), 8% were nulliparous throughout the follow-up period, at which point the youngest were 38 years old. Nulliparous and parous women had similar age distributions, but nulliparous women were less likely to smoke or consume alcohol and more likely to participate in vigorous physical activity (**Table 1**). Nulliparous women were slightly more likely to be obese and had greater levels of nonparticipation and missing data.

**TABLE 1.** Description of covariates at the individual and observational level based on final parity status among HUNT2 and HUNT3 study participants born between 1940 and 1974 (*n* = 21,312 women)

	Final Parity Status	
	Nulliparous	Parous
Women (*n*)	1,625	19,687
Birth year [median (IQR)]	1959 (1951–1967)	1958 (1951–1966)
Smoking status at the age of 20 years [*n* (%)]		
Never smoked daily	919 (57)	9,043 (46)
Ever smoked daily	653 (40)	10,166 (52)
Not reported	53 (3)	478 (2)
Education [*n* (%)]		
Lower secondary	309 (19)	3,164 (16)
Upper secondary	672 (41)	9,185 (47)
Tertiary	604 (37)	7,244 (37)
Missing	40 (2)	94 (0)
Family history of CVD [*n* (%)]	504 (31)	6,611 (34)
HUNT exam participation [*n* (%)]		
Only HUNT2	751 (46)	6,827 (35)
Only HUNT3	288 (18)	3,333 (17)
Both HUNT2 and 3	586 (36)	9,527 (48)
Age in years at first birth [median (IQR)]	N/A	23 (20–26)
Births [*n* (%)]		
1	N/A	2,171 (11)
2	N/A	8,804 (45)
≥3	N/A	8,712 (44)
Breastfeeding length of first birth [*n* (%)]		
Did not breastfeed	N/A	1,120 (6)
<3 months	N/A	3,086 (16)
3-6 months	N/A	5,503 (28)
>6 months	N/A	7,462 (38)
Missing	N/A	2,516 (13)
Preterm first birth [*n* (%)]	N/A	1,187 (6)
Observations*^a^* (*n*)	3,329	31,743
BMI at HUNT exam [*n* (%)]		
<25 kg/m^2^	1,674 (50)	15,728 (50)
25–29.9 kg/m^2^	963 (29)	10,826 (34)
≥30 kg/m^2^	665 (20)	5,136 (16)
Not available	27 (1)	53 (0)
Alcohol consumption [*n* (%)]		
<1 glasses per 2 weeks	1,162 (35)	9,277 (29)
1–4 glasses per 2 weeks	1,167 (35)	14,797 (47)
≥5 glasses per 2 weeks	932 (28)	7,221 (23)
Missing	68 (2)	448 (1)
Vigorous physical activity [*n* (%)]		
<1 h per week	1,669 (50)	18,327 (58)
≥1 h per week	1,183 (36)	9,921 (31)
Missing	477 (14)	3,495 (11)
Oral contraceptive use [*n* (%)]		
Nonuser	2,024 (61)	22,716 (72)
Current user	564 (17)	2,961 (9)
Missing	741 (22)	6,066 (19)

a Observations reflect HUNT survey periods. Individual women who participated in both HUNT2 and HUNT3 contributed two observations.

**Fig. 2.**
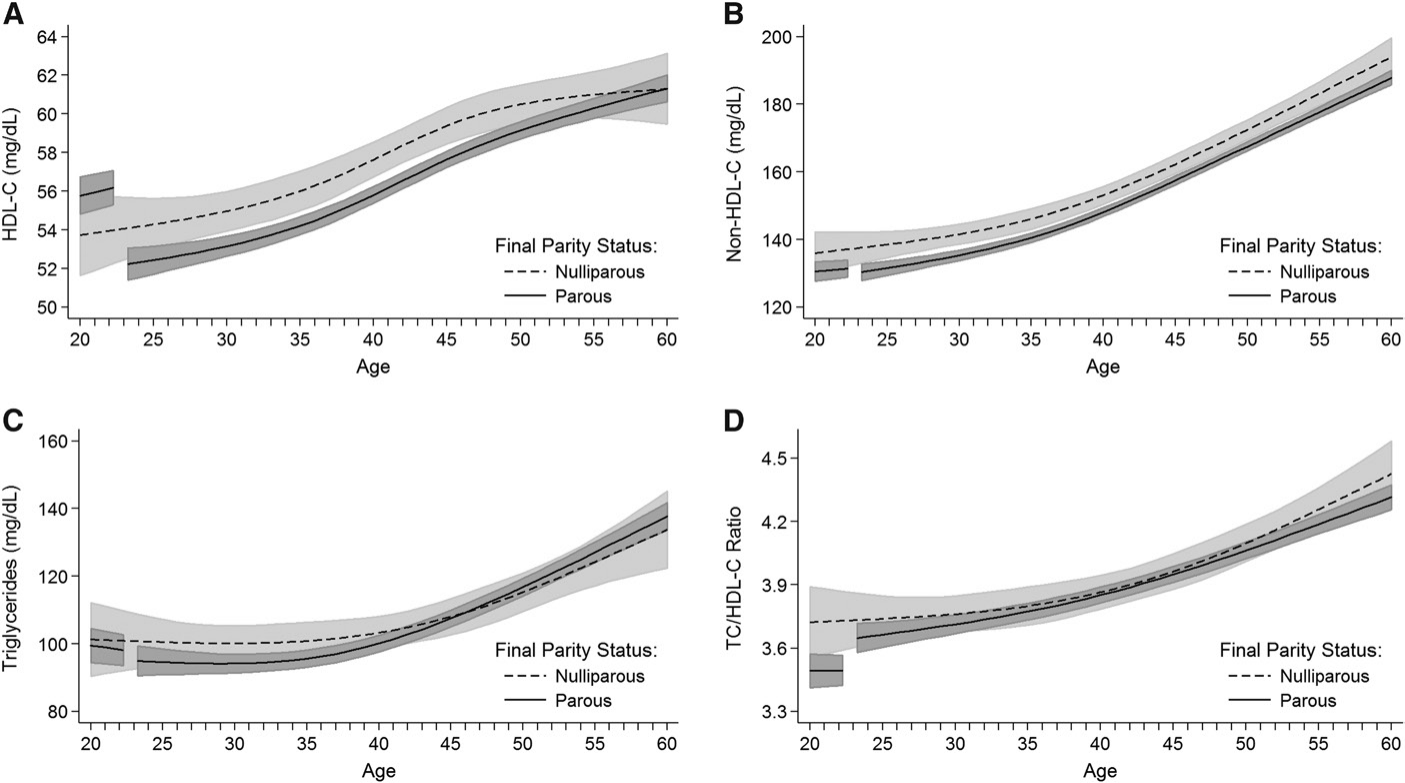
Predicted lipid trajectories and 95% CIs based on final parity status for (A) HDL-C, (B) non-HDL-C, (C) triglycerides, and (D) TC-HDL-C ratio. Estimates for parous women are predicted for women with a hypothetical first birth at the age of 23 years. Gaps represent pregnancy and 3 months postpartum. Predictions are at the mean level for the following covariates: HUNT survey, time since last meal, education, smoking initiation by the age of 20 years, and family history of cardiovascular disease.

Predicted life-course lipid trajectories, based on multivariable models, suggested higher HDL-C levels among parous women before first birth compared with nulliparous women (**Fig. 2A**), with a difference of 2.0 mg/dl (95% CI: −0.1, 4.2) at the age of 20 years (**Table 2**). However, the HDL-C levels of parous women dropped at first pregnancy and thereafter were lower or equal to those of nulliparous women (Table 2). HDL-C in parous women after their first birth never returned to the same levels that would be predicted given the observed prepregnancy slope (Fig. 2A). Using the full population of parous women, the estimated short-term drop in HDL-C after first birth was −4.2 mg/dl (95% CI: −5.0, −3.3) (model 1, **Table 3**), representing a 7% decrease in HDL-C compared with average prepregnancy levels. This decrease dropped to −3.0 mg/dl (95% CI: −4.2, −1.8) after adjusting for updated covariates, including BMI (model 2, Table 3).

**TABLE 2.** Predicted lipid levels by age based on final parity status for HUNT2 and HUNT3 participants born between 1940 and 1974 (*n* = 21,312 women)

Lipids*^a^*	Nulliparous		Parous with First Birth at the Age of 23 Years		Difference (Parous − Nulliparous)		
	Estimate	95% CI	Estimate	95% CI	Estimate	95% CI	*P*
HDL-C (mg/dl)							
20 years	53.7	51.6, 55.8	55.8	54.8, 56.7	2.0	−0.1, 4.2	0.06
30 years	55.0	54.0, 56.0	53.1	52.6, 53.7	−1.8	−2.8, -0.8	<0.001
40 years	57.6	56.7, 58.5	55.8	55.4, 56.2	−1.8	−2.7, -1.0	<0.001
50 years	60.5	59.5, 61.5	59.1	58.7, 59.6	−1.4	−2.3, −0.4	0.006
60 years	61.3	59.5, 63.1	61.3	60.6, 62.0	0.0	−1.8, 1.8	0.99
Non-HDL-C (mg/dl)							
20 years	136.0	129.9, 142.1	130.6	127.8, 133.4	−5.4	−11.6, 0.7	0.09
30 years	141.6	138.7, 144.5	135.4	133.9, 136.9	−6.2	−9.0, −3.3	<0.001
40 years	153.1	150.5, 155.7	148.2	146.9, 149.5	−4.9	−7.4, −2.5	<0.001
50 years	172.5	169.6, 175.4	167.6	166.3, 169.0	−4.9	−7.7, −2.1	<0.001
60 years	194.0	188.5, 199.5	188.0	185.9, 190.0	−6.1	−11.6, -0.6	0.03
Triglycerides (mg/dl)							
20 years	101.3	90.4, 112.2	99.6	94.5, 104.6	−1.7	−12.7, 9.2	0.76
30 years	100.2	95.0, 105.3	94.3	91.6, 97.1	−5.9	−10.9, −0.9	0.02
40 years	103.3	98.6, 108.1	100.2	97.8, 102.7	−3.1	−7.6, 1.4	0.18
50 years	115.3	109.6, 120.9	116.9	114.3, 119.5	1.6	−3.8, 7.0	0.56
60 years	133.8	122.5, 145.2	137.9	133.8, 141.9	4.0	−7.4, 15.5	0.49
TC/HDL-C ratio							
20 years	3.7	3.6, 3.9	3.5	3.4, 3.6	−0.23	−0.40, −0.06	0.008
30 years	3.8	3.7, 3.8	3.7	3.7, 3.8	−0.05	−0.13, 0.04	0.28
40 years	3.9	3.8, 3.9	3.9	3.8, 3.9	−0.01	−0.09, 0.07	0.78
50 years	4.1	4.0, 4.2	4.1	4.0, 4.1	−0.03	−0.12, 0.05	0.45
60 years	4.4	4.3, 4.6	4.3	4.3, 4.4	−0.11	−0.26, 0.05	0.17

a Adjusted for participant’s age at measurement, HUNT survey, time since last meal, education, smoking initiation by the age of 20 years, and family history of CVD.

**TABLE 3.** Differences in lipid levels from prepregnancy to after first birth by breastfeeding length for parous HUNT2 and HUNT3 study participants (*n* = 22,047 women)

	Model 1*^a^*			Model 2*^b^*		
	Estimate	95% CI	*P*	Estimate	95% CI	*P*
HDL-C (mg/dl)						
All parous women	−4.2	−5.0, −3.3	<0.001	−3.0	−4.2, −1.8	<0.001
By breastfeeding length						
Did not breastfeed	−6.2	−7.3, −5.1	<0.001	−3.9	−5.3, −2.5	<0.001
Breastfed <3 months	−5.2	−6.1, −4.3	<0.001	−3.5	−4.7, −2.2	<0.001
Breastfed 3-6 months	−4.0	−4.9, −3.1	<0.001	−2.7	−3.9, −1.5	<0.001
Breastfed >6 months	−3.8	−4.6, −2.9	<0.001	−2.9	−4.1, −1.7	<0.001
Non-HDL-C (mg/dl)						
All parous women	−0.4	−2.8, 2.1	0.77	1.1	−2.3, 4.5	0.53
By breastfeeding length						
Did not breastfeed	5.6	2.3, 8.8	<0.001	3.7	−0.3, 7.7	0.07
Breastfed <3 months	2.2	−0.5, 5.0	0.11	2.1	−1.5, 5.7	0.25
Breastfed 3-6 months	0.5	−2.0, 3.1	0.67	1.5	−2.0, 5.0	0.39
Breastfed >6 months	−1.9	−4.4, 0.5	0.13	0.3	−3.1, 3.8	0.85
Triglycerides (mg/dl)						
All parous women	−3.6	−7.8, 0.7	0.10	1.8	−3.8, 7.5	0.52
By breastfeeding length						
Did not breastfeed	9.7	4.1, 15.4	<0.001	7.5	0.8, 14.2	0.03
Breastfed <3 months	1.9	−2.9, 6.7	0.43	3.8	−2.2, 9.8	0.22
Breastfed 3-6 months	−2.6	−7.1, 1.9	0.25	1.8	−4.0, 7.7	0.54
Breastfed >6 months	−6.6	−10.9, −2.3	0.003	0.7	−5.1, 6.4	0.82
TC/HDL-C ratio						
All parous women	0.18	0.11, 0.25	<0.001	0.16	0.06, 0.25	<0.001
By breastfeeding length						
Did not breastfeed	0.43	0.33, 0.52	<0.001	0.27	0.16, 0.39	<0.001
Breastfed <3 months	0.29	0.21, 0.37	<0.001	0.21	0.11, 0.31	<0.001
Breastfed 3-6 months	0.19	0.12, 0.26	<0.001	0.15	0.06, 0.25	0.002
Breastfed >6 months	0.12	0.06, 0.19	<0.001	0.13	0.04, 0.23	0.006

Models include all parous women without restricting to participants born between 1940 and 1974, as in Fig. 2 and Table 2 comparisons with nulliparous women.

a Adjusted for participant’s age at measurement, HUNT survey, time since last meal, education, smoking initiation by the age of 20 years, and family history of CVD.

b Additionally adjusted for updated BMI, updated alcohol use, updated physical activity, updated oral contraceptive use, maternal age at first birth, and preterm first birth.

Non-HDL-C was lower in parous compared with nulliparous women (Fig. 2B, Table 2), with no meaningful changes at first birth (Table 3). Triglycerides were similar in the two groups (Fig. 2C, Table 2) and did not meaningfully change after first birth. As expected, given lower non-HDL-C levels and higher HDL-C levels, the TC/HDL-C ratio was lower in parous women before their first birth compared with nulliparous women [−0.23 units (95% CI: −0.40, −0.06)] (Table 2). After first birth, this ratio increased by an estimated 0.18 units (95% CI: 0.11, 0.25) among parous women (model 1, Table 3). In the decades after first birth, the TC/HDL-C ratio for parous women was similar to the ratio for nulliparous women of the same age, with no indication of a return to the lower, more favorable ratio experienced by parous women before their first birth (Fig. 2D, Table 2). Changes in lipid levels before to after first birth did not differ based on maternal age at first birth or gestation length of first birth (results not shown).

The majority of parous women breastfed for at least 3 months (Table 1), and longer breastfeeding times were associated with less adverse changes in all lipid levels (Table 3). The changes in lipid analyte levels before to after first birth were moderated by breastfeeding length in all models included in Table 3 (*P* < 0.01; results not shown). However, women who breastfed >6 months after first birth still experienced a decrease in HDL-C and an increase in the TC/HDL-C ratio after first birth, even after adjusting for updated covariates (model 2, Table 3). After restricting analyses to women with two or more births, we found similar changes in HDL-C and the TC/HDL-C ratio after first birth, but changes after subsequent births were smaller in magnitude (**Table 4**).

**TABLE 4.** Differences in lipid levels from prepregnancy to after birth by birth order for parous HUNT2 and HUNT3 study participants with at least two births*^a^*

	Women with 2+ Births (*n* = 18,956)			Women with 3+ Births (*n* = 8,976)		
	Estimate	95% CI	*P*	Estimate	95% CI	*P*
HDL-C (mg/dl)						
First birth	−3.6	−4.8, −2.5	<0.001	−2.7	−4.8, −0.6	0.01
Second birth	−1.6	−2.4, −0.7	<0.001	−2.0	−3.4, −0.5	0.008
Third birth				−2.2	−3.2, −1.3	<0.001
Non-HDL-C (mg/dl)						
First birth	2.7	−0.6, 6.1	0.11	3.6	−2.4, 9.7	0.24
Second birth	−1.4	−3.9, 1.1	0.27	0.8	−3.6, 5.1	0.73
Third birth				0.2	−2.6, 3.1	0.86
Triglycerides (mg/dl)						
First birth	−0.3	−6.1, 5.5	0.92	5.9	−4.2, 16.0	0.25
Second birth	−2.2	−6.6, 2.3	0.34	0.1	−7.4, 7.6	0.98
Third birth				0.1	−5.0, 5.2	0.98
TC/HDL-C ratio						
First birth	0.22	0.12, 0.31	<0.001	0.18	0.02, 0.34	0.03
Second birth	0.03	−0.05, 0.10	0.49	0.09	−0.03, 0.21	0.12
Third birth				0.08	−0.00, 0.16	0.05

a Adjusted for participant’s age at measurement, HUNT survey, time since last meal, education, smoking initiation by the age of 20 years, and family history of CVD.

Sensitivity analyses among the subset of women with repeated lipid measurements yielded similar predicted lipid trajectories, although with wider CIs due to the lower statistical power (supplemental Fig. S3). Women with a birth between HUNT2 and HUNT3 experienced a decrease in HDL-C and an increase in TC/HDL-C compared with women who did not give birth during the ∼11 year period, with a magnitude similar to associations seen in the main analysis (supplemental Table S2). Lipid trajectories were also similar after adjusting for menopause and hormone replacement therapy use (supplemental Fig. S4).

## DISCUSSION

We found that women who bore children had more positive lipid profiles before first birth compared with women who remained nulliparous throughout life. However, this advantage changed at first birth, when new mothers had a decrease in HDL-C and an increase in the TC/HDL-C ratio compared with nulliparous women. Our findings suggest that adverse changes in HDL-C and TC/HDL-C associated with pregnancy persist across a woman’s life course. In addition, the specificity of the timing of the change in these lipid profiles suggests that pregnancy itself causes the drop in HDL. Adverse changes after pregnancy were present after adjusting for a variety of behavioral changes associated with a first birth, including higher BMI, lower alcohol use, less physical activity, and less oral contraceptive use. Although longer breastfeeding appeared to moderate adverse changes in HDL-C and the TC/HDL-C ratio after pregnancy, women who breastfed for >6 months still experienced worse lipid profiles postpartum compared with prepregnancy levels.

The magnitude of the drop in HDL-C associated with first pregnancy of −4.2 mg/dl is consistent with previous studies that examined short-term changes in lipids from prepregnancy to postpartum (3, 6, 7, 9). In the longitudinal Coronary Artery Risk Development in Young Adults (CARDIA) Study, HDL-C differed by −3 to −4 mg/dl over an interval of 2–8 years for women who had a first birth during the interval compared with a nulliparous reference group (6, 9). Using a study design similar to CARDIA, adolescents in the National Heart Lung and Blood Institute’s Growth and Health Study who had a first birth during a 10 year interval had a −4 mg/dl decrease in HDL-C compared with a nulliparous reference group. The similarity of estimates in an adolescent population is consistent with our finding that the changes in lipids associated with pregnancy did not differ based on maternal age at first birth. These studies similarly found that the changes after pregnancy were specific to HDL-C (3, 6–9), although one study found elevated triglycerides postpartum (7). Our study obtained similar results despite different strengths and weaknesses compared with previous studies. For instance, CARDIA had repeated measurements for all participants but examined changes only up to 10 years, while our study expanded measurement up to 40 years postpartum but included a mixture of cross-sectional and longitudinal data.

Our study is the first to report the association between parity and the TC/HDL-C ratio, which has been found to have a stronger independent predictive power for CVD over its component lipid measures (18, 22, 23, 40), providing a more holistic atherogenic index. Based on this measure, while parous women lose their initial advantage of high HDL-C and low non-HDL-C after their first birth, their long-term TC/HDL-C trajectory is no worse than that of nulliparous women.

The use of a nulliparous comparison group is a strength of this study, as it allowed us to distinguish the change after pregnancy from any background age or secular trends in our population. Previous cross-sectional studies that compared nulliparous and parous women postpartum have generally found lower lipid levels among parous women after they deliver, consistent with our findings (15, 41, 42). Our study suggests that the findings from these cross-sectional studies may have underestimated differences related to pregnancy, as nulliparous women had more atherogenic lipid profiles compared with parous women before their first birth. Given the less atherogenic lipid profiles among parous women earlier in life, it is striking that they experienced an almost identical TC/HDL-C ratio and a significantly worse HDL-C profile from postpartum through the age of 50 years.

Our findings that breastfeeding may moderate the short-term adverse changes in HDL-C levels associated with pregnancy is consistent with findings from the CARDIA study (17). However, the CARDIA study found more dramatic associations between breastfeeding and the change in HDL-C levels after birth, with those who breastfed 0 to <3 months having a −7.3 mg/dl drop in HDL-C compared with only a −1.3 mg/dl drop among women who breastfed ≥3 months. We were able to control for potential confounders not captured in the CARDIA study, including alcohol use, physical activity, and preterm first birth, and found that women who breastfed >6 months still experienced a drop in HDL-C of −2.9 mg/dl compared with a drop of −3.9 mg/dl among those who did not breastfeed. Despite controlling for several covariates, there is still the potential for confounding to explain some or all of the differences we observed across categories of breastfeeding length. In Norway, there are particularly high breastfeeding rates (43) and fewer cultural barriers (44), potentially leading to less confounding by socioeconomic factors than in other contexts; however, the minority of women who do not breastfeed are still likely a highly selected population. While breastfeeding is associated with higher HDL-C levels before weaning (11–14) and a more rapid return of triglycerides to prepregnancy levels (16), it is also unclear whether these findings would extend over a longer period of time (15, 45). While we were able to quantify the association of breastfeeding duration with the change in lipid levels before and after pregnancy, we lacked sufficient data to examine long-term lipid trajectories by breastfeeding history.

A limitation of this study is the lack of repeated measurements for all women. By including women who contributed only one measurement, we were able to examine a much larger span of time postpartum than had previous studies. However, such trajectories, constructed of both longitudinal and cross-sectional data, may not reflect an individual woman’s trajectory across her life course, as there is the potential for secular trends to influence the shape of trajectories. Such concerns should not affect the comparisons of parous and nulliparous women, who experienced the same secular trends. We also found similar results after restricting our analysis to the 47% of women with repeated measurements, suggesting that it is reasonable to estimate within-woman changes from these trajectories.

Another limitation of our study is the nonfasting collection of lipids. Although we controlled for the time since last meal in all analyses, we lacked information on the content of the last meal, which may have affected triglyceride measurements but likely had a minimal effect on other lipid measures (19, 46). This source of error combined with the relatively large variation in triglyceride measurements (47) may have led to wider CIs for triglycerides compared with other lipid measures and could have obscured the effects of pregnancy. Of note, nonfasting lipids are predictive of CVD risk (46, 48–50), and thus their association with pregnancy is relevant for understanding early subclinical CVD risk. Although nonfasting lipid measurement limited us from accurately measuring LDL-C, non-HDL-C is considered as good as or a better predictor of CVD risk (23, 51, 52). Lipid measurement methods also differed somewhat between HUNT2 and HUNT3 exams, which could have influenced the shape of our life-course lipid trajectories. Although we did not find evidence of systematically higher or lower lipid levels by HUNT exam, we controlled for HUNT survey occasion in all analyses, which would adjust our estimates of within-woman change for any differences in measurement methods.

We also were unable to adjust for statin use, which may have altered the shape of lipid trajectories. However, rates of use were low among 20 to 60 year olds during our study period (53), and usage is likely to have influenced the trend in the nulliparous and parous groups equally. Menopause transition or hormone replacement therapy use may also have affected lipid trajectories at older ages; however, controlling for these variables did not meaningfully change study findings in sensitivity analyses. We were also unable to control for either diet or sleep duration, which may change after pregnancy and might be considered one of the pathways through which parity affects lipid levels. However, a previous study did not find that diet explained a large portion of the drop in HDL-C after pregnancy (6). While our study location in Nord-Trøndelag county is fairly representative of Norway (26), the population is ethnically homogenous, which may limit the generalizability of our findings. Previous studies found similar differences in lipids associated with pregnancy among white and black women (6, 7), suggesting that any effects of first birth on lipids are similar across a range of contexts.

As with other studies on this topic, we found that the difference in HDL-C after pregnancy was still present after adjusting for changes in BMI and a variety of lifestyle factors postpartum (6, 7, 9, 10). The mechanism for the drop in HDL-C is only speculative, but one hypothesis is that it is related to hormonal changes postpartum. Parous women have tended to have lower levels of estrogen compared with nulliparous women in most, but not all, studies, including those extending over multiple years postpartum (54–59). Estrogen is known to be positively associated with HDL-C levels, suggesting estrogen suppression after pregnancy may be implicated. Declines in HDL-C with pregnancy have been shown to differ based on the apoE phenotype, suggesting an interaction between genetic and hormonal components (60). In addition, studies have suggested that changes in HDL may differ by subclass, with larger HDL-2 particles declining after pregnancy more than other, smaller subclasses of HDL-C, leading to a redistribution toward a smaller particle size postpartum (9, 61). Only one previous study (3) distinguished between changes in lipids after first and later births; this study found diminishing changes after subsequent births. Mankuta et al. (3) also noted that the rise in HDL-C during pregnancy was smaller in each subsequent birth. Further studies to elucidate this mechanism are needed to explain why the postpregnancy drop in HDL-C depends on parity.

These findings suggest that pregnancy is associated with a lasting adverse change in HDL-C that sets parous women on a more atherogenic trajectory than they had before pregnancy. Extrapolating from Hartz et al. (53), the 4.2 mg/dl decrease in HDL-C with first birth would be associated with a 7% increase in the rate of coronary heart disease, while the 1.4 mg/dl decrease in HDL-C at the age of 50 years among parous women would be associated with a 2% increase in coronary heart disease rates, suggesting the short- and long-term changes in HDL-C associated with pregnancy are also associated with relatively small changes in CVD risk. While HDL-C is strongly predictive of CVD risk (62, 63), recent findings suggest that raising HDL-C levels may not causally lead to improvements in cardiovascular endpoints (56). Characterizing the effect of pregnancy on more direct measures of HDL function such as cholesterol efflux capacity (64, 65), the ability to remove cholesterol from cells, would be useful in future studies.

## Supplementary Material

Supplemental Data

## References

[1] Di Angelantonio E., Sarwar N., Perry P., Kaptoge S., Ray K. K., Thompson A., Wood A. M., Lewington S., Sattar N., Packard C. J., 2009 Major lipids, apolipoproteins, and risk of vascular disease. JAMA. 302: 1993–2000. 19903920 10.1001/jama.2009.1619 PMC3284229

[2] Rosenson R. S. 2016 The high-density lipoprotein puzzle: why classic epidemiology, genetic epidemiology, and clinical trials conflict? Arterioscler. Thromb. Vasc. Biol. 36: 777–782. 26966281 10.1161/ATVBAHA.116.307024

[3] Mankuta D., Elami-Suzin M., Elhayani A., and Vinker S. 2010 Lipid profile in consecutive pregnancies. Lipids Health Dis. 9: 58. 20525387 10.1186/1476-511X-9-58 PMC2904773

[4] Knopp R. H., Warth M. R., Charles D., Childs M., Li J. R., Mabuchi H., and Van Allen M. I. 1986 Lipoprotein metabolism in pregnancy, fat transport to the fetus, and the effects of diabetes. Biol. Neonate. 50: 297–317. 3542067 10.1159/000242614

[5] Potter J. M., and Nestel P. J. 1979 The hyperlipidemia of pregnancy in normal and complicated pregnancies. Am. J. Obstet. Gynecol. 133: 165–170. 217273 10.1016/0002-9378(79)90469-1

[6] Gunderson E. P., Lewis C. E., Murtaugh M. A., Quesenberry C. P., Smith West D., and Sidney S. 2004 Long-term plasma lipid changes associated with a first birth: the Coronary Artery Risk Development in Young Adults study. Am. J. Epidemiol. 159: 1028–1039. 15155287 10.1093/aje/kwh146 PMC4107869

[7] Gunderson E. P., Schreiber G., Striegel-Moore R., Hudes M., Daniels S., Biro F. M., and Crawford P. B. 2012 Pregnancy during adolescence has lasting adverse effects on blood lipids: a 10-year longitudinal study of black and white females. J. Clin. Lipidol. 6: 139–149. 22385547 10.1016/j.jacl.2011.12.004 PMC3376747

[8] van Stiphout W. A., Hofman A., and Bruijn A. M. D. 1987 Serum lipids in young women before, during, and after pregnancy. Am. J. Epidemiol. 126: 922–928. 3661539 10.1093/oxfordjournals.aje.a114729

[9] Lewis C. E., Funkhouser E., Raczynski J. M., Sidney S., Bild D. E., and Howard B. V. 1996 Adverse effect of pregnancy on high density lipoprotein (HDL) cholesterol in young adult women. The CARDIA Study. Coronary Artery Risk Development in Young Adults. Am. J. Epidemiol. 144: 247–254. 8686693 10.1093/oxfordjournals.aje.a008919

[10] Skilton M. R., Bonnet F., Begg L. M., Juonala M., Kähönen M., Lehtimäki T., Viikari J. S. A., and Raitakari O. T. 2010 Childbearing, child-rearing, cardiovascular risk factors, and progression of carotid intima-media thickness. Stroke. 41: 1332–1337. 20538698 10.1161/STROKEAHA.110.579219

[11] Knopp R. H., Walden C. E., Wahl P. W., Bergelin R., Chapman M., Irvine S., and Albers J. J. 1985 Effect of postpartum lactation on lipoprotein lipids and apoproteins. J. Clin. Endocrinol. Metab. 60: 542–547. 3972965 10.1210/jcem-60-3-542

[12] Erkkola R., Viikari J., Irjala K., and Solakivi-Jaakkola T. 1986 One-year follow-up of lipoprotein metabolism after pregnancy. Biol. Res. Pregnancy Perinatol. 7: 47–51. 3730470

[13] Kallio M. J., Siimes M. A., Perheentupa J., Salmenperä L., and Miettinen T. A. 1992 Serum cholesterol and lipoprotein concentrations in mothers during and after prolonged exclusive lactation. Metabolism. 41: 1327–1330. 1461138 10.1016/0026-0495(92)90103-h

[14] Kjos S. L., Henry O., Lee R. M., Buchanan T. A., and Mishell D. R. 1993 The effect of lactation on glucose and lipid metabolism in women with recent gestational diabetes. Obstet. Gynecol. 82: 451–455. 8355952

[15] Natland S. T., Nilsen T. I. L., Midthjell K., Andersen L. F., and Forsmo S. 2012 Lactation and cardiovascular risk factors in mothers in a population-based study: the HUNT-study. Int. Breastfeed. J. 7: 8. 22713515 10.1186/1746-4358-7-8 PMC3489591

[16] Darmady J. M., and Postle A. D. 1982 Lipid metabolism in pregnancy. Br. J. Obstet. Gynaecol. 89: 211–215. 7066258 10.1111/j.1471-0528.1982.tb03616.x

[17] Gunderson E. P., Lewis C. E., Wei G. S., Whitmer R. A., Quesenberry C. P., and Sidney S. 2007 Lactation and changes in maternal metabolic risk factors. Obstet. Gynecol. 109: 729–738. 17329527 10.1097/01.AOG.0000252831.06695.03 PMC2930880

[18] Manickam P., Rathod A., Panaich S., Hari P., Veeranna V., Badheka A., Jacob S., and Afonso L. 2011 Comparative prognostic utility of conventional and novel lipid parameters for cardiovascular disease risk prediction: do novel lipid parameters offer an advantage? J. Clin. Lipidol. 5: 82–90. 21392721 10.1016/j.jacl.2010.12.001

[19] Mora S., Otvos J. D., Rifai N., Rosenson R. S., Buring J. E., and Ridker P. M. 2009 Lipoprotein particle profiles by nuclear magnetic resonance compared with standard lipids and apolipoproteins in predicting incident cardiovascular disease in women. Circulation. 119: 931–939. 19204302 10.1161/CIRCULATIONAHA.108.816181 PMC2663974

[20] McQueen M. J., Hawken S., Wang X., Ounpuu S., Sniderman A., Probstfield J., Steyn K., Sanderson J. E., Hasani M., Volkova E., 2008 Lipids, lipoproteins, and apolipoproteins as risk markers of myocardial infarction in 52 countries (the INTERHEART study): a case-control study. Lancet. 372: 224–233. 18640459 10.1016/S0140-6736(08)61076-4

[21] Kastelein J. J. P., van der Steeg W. A., Holme I., Gaffney M., Cater N. B., Barter P., Deedwania P., Olsson A. G., Boekholdt S. M., Demicco D. A., 2008 Lipids, apolipoproteins, and their ratios in relation to cardiovascular events with statin treatment. Circulation. 117: 3002–3009. 18519851 10.1161/CIRCULATIONAHA.107.713438

[22] Ingelsson E., Schaefer E. J., Contois J. H., McNamara J. R., Sullivan L., Keyes M. J., Pencina M. J., Schoonmaker C., Wilson P. W. F., D’Agostino R. B., 2007 Clinical utility of different lipid measures for prediction of coronary heart disease in men and women. JAMA. 298: 776–785. 17699011 10.1001/jama.298.7.776

[23] Ridker P. M., Rifai N., Cook N. R., Bradwin G., and Buring J. E. 2005 Non-HDL cholesterol, apolipoproteins A-I and B100, standard lipid measures, lipid ratios, and CRP as risk factors for cardiovascular disease in women. JAMA. 294: 326–333. 16030277 10.1001/jama.294.3.326

[24] Natarajan S., Glick H., Criqui M., Horowitz D., Lipsitz S. R., and Kinosian B. 2003 Cholesterol measures to identify and treat individuals at risk for coronary heart disease. Am. J. Prev. Med. 25: 50–57. 12818310 10.1016/s0749-3797(03)00092-8

[25] Krokstad S., Langhammer A., Hveem K., Holmen T. L., Midthjell K., Stene T. R., Bratberg G., Heggland J., and Holmen J. 2013 Cohort profile: the HUNT Study, Norway. Int. J. Epidemiol. 42: 968–977. 22879362 10.1093/ije/dys095

[26] Holmen J., Midthjell K., Krüger Ø., Langhammer A., Holmen T. L., Bratberg G. H., Vatten L., and Lund-Larsen P. G. 2003 The Nord-Trøndelag Health Study 1995–97 (HUNT 2): Objectives, contents, methods and participation. Nor. Epidemiol. 13: 19–32.

[27] Irgens L. M. 2000 The Medical Birth Registry of Norway. Epidemi­ological research and surveillance throughout 30 years. Acta Obstet. Gynecol. Scand. 79: 435–439. 10857866

[28] Friedewald W. T., Levy R. I., and Fredrickson D. S. 1972 Estimation of the concentration of low-density lipoprotein cholesterol in plasma, without use of the preparative ultracentrifuge. Clin. Chem. 18: 499–502. 4337382

[29] Scharnagl H., Nauck M., Wieland H., and März W. 2001 The Friedewald formula underestimates LDL cholesterol at low concentrations. Clin. Chem. Lab. Med. 39: 426–431. 11434393 10.1515/CCLM.2001.068

[30] Jun K. R., Park H-I., Chun S., Park H., and Min W-K. 2008 Effects of total cholesterol and triglyceride on the percentage difference between the low-density lipoprotein cholesterol concentration measured directly and calculated using the Friedewald formula. Clin. Chem. Lab. Med. 46: 371–375. 18254713 10.1515/CCLM.2008.064

[31] Lindsey C. C., Graham M. R., Johnston T. P., Kiroff C. G., and Freshley A. 2004 A clinical comparison of calculated versus direct measurement of low-density lipoprotein cholesterol level. Pharmacotherapy. 24: 167–172. 14998216 10.1592/phco.24.2.167.33142

[32] Statistics Norway. 1998 Standard classification of occupations. Accessed July 15, 2016, at https://www.ssb.no/a/publikasjoner/pdf/nos_c521/nos_c521.pdf.

[33] Harrell F. E. 2010 Springer, New York.

[34] Kim J. J., and Choi Y. M. 2013 Dyslipidemia in women with polycystic ovary syndrome. Obstet. Gynecol. Sci. 56: 137–142. 24327994 10.5468/ogs.2013.56.3.137 PMC3784112

[35] Mahalingaiah S., Sun F., Cheng J. J., Chow E. T., Lunetta K. L., and Murabito J. M. 2017 Cardiovascular risk factors among women with self-reported infertility. Fertil. Res. Pract. 3: 7. 28620545 10.1186/s40738-017-0034-0 PMC5424365

[36] van Buuren S. 2007 Multiple imputation of discrete and continuous data by fully conditional specification. Stat. Methods Med. Res. 16: 219–242. 17621469 10.1177/0962280206074463

[37] Li K-H., Meng X-L., Raghunathan T. E., and Rubin D. B. 1991 Significance levels from repeated p-values with multiply-imputed data. Stat. Sin. 1: 65–92.

[38] Imbens G. W., and Wooldridge J. M. 2009 Recent developments in the econometrics of program evaluation. J. Econ. Lit. 47: 5–86.

[39] Rasbash J., Charlton C., Browne W. J., Healy M., and Cameron B. 2009 MLwiN Ver. 2.34. Centre for Multilevel Modelling, University of Bristol, Bristol, UK.

[40] Hsia S. H., Pan D., Berookim P., and Lee M. L. 2006 A population-based, cross-sectional comparison of lipid-related indexes for symptoms of atherosclerotic disease. Am. J. Cardiol. 98: 1047–1052. 17027569 10.1016/j.amjcard.2006.05.024

[41] Lv H., Yang X., Zhou Y., Wu J., Liu H., Wang Y., Pan Y., and Xia Y. 2016 Parity and serum lipid levels: a cross-sectional study in chinese female adults. Sci. Rep. 6: 33831. 10.1038/srep33831 PMC5028753 27645134

[42] Hardy R., Lawlor D. A., Black S., Wadsworth M. E. J., and Kuh D. 2007 Number of children and coronary heart disease risk factors in men and women from a British birth cohort. BJOG. 114: 721–730. 17516964 10.1111/j.1471-0528.2007.01324.x

[43] Lande B., Andersen L. F., Baerug A., Trygg K. U., Lund-Larsen K., Veierød M. B., and Bjørneboe G. E. A. 2003 Infant feeding practices and associated factors in the first six months of life: the Norwegian infant nutrition survey. Acta Paediatr. 92: 152–161. 12710639 10.1111/j.1651-2227.2003.tb00519.x

[44] Australian Government Department of Health and Ageing. Norway—the WHO code and breastfeeding: an international comparative overview. Accessed April 5, 2017, at http://www.health.gov.au/internet/publications/publishing.nsf/Content/int-comp-whocode-bf-init~int-comp-whocode-bf-init-ico~int-comp-whocode-bf-init-ico-norway.

[45] Stuebe A. M., Schwarz E. B., Grewen K., Rich-Edwards J. W., Michels K. B., Foster E. M., Curhan G., and Forman J. 2011 Duration of lactation and incidence of maternal hypertension: a longitudinal cohort study. Am. J. Epidemiol. 174: 1147–1158. 21997568 10.1093/aje/kwr227 PMC3246687

[46] Langsted A., Freiberg J. J., and Nordestgaard B. G. 2008 Fasting and nonfasting lipid levels. Circulation. 118: 2047–2056. 18955664 10.1161/CIRCULATIONAHA.108.804146

[47] Mogadam M., Ahmed S. W., Mensch A. H., and Godwin I. D. 1990 Within-person fluctuations of serum cholesterol and lipoproteins. Arch. Intern. Med. 150: 1645–1648. 2383159

[48] Mora S., Rifai N., Buring J. E., and Ridker P. M. 2008 Fasting compared with nonfasting lipids and apolipoproteins for predicting incident cardiovascular events. Circulation. 118: 993–1001. 18711012 10.1161/CIRCULATIONAHA.108.777334 PMC2574817

[49] Nordestgaard B. G., Benn M., Schnohr P., and Tybjaerg-Hansen A. 2007 Nonfasting triglycerides and risk of myocardial infarction, ischemic heart disease, and death in men and women. JAMA. 298: 299–308. 17635890 10.1001/jama.298.3.299

[50] Bansal S., Buring J. E., Rifai N., Mora S., Sacks F. M., and Ridker P. M. 2007 Fasting compared with nonfasting triglycerides and risk of cardiovascular events in women. JAMA. 298: 309–316. 17635891 10.1001/jama.298.3.309

[51] Liu J., Sempos C., Donahue R. P., Dorn J., Trevisan M., and Grundy S. M. 2005 Joint distribution of non-HDL and LDL cholesterol and coronary heart disease risk prediction among individuals with and without diabetes. Diabetes Care. 28: 1916–1921. 16043732 10.2337/diacare.28.8.1916

[52] Cui Y., Blumenthal R. S., Flaws J. A., Whiteman M. K., Langenberg P., Bachorik P. S., and Bush T. L. 2001 Non-high-density lipoprotein cholesterol level as a predictor of cardiovascular disease mortality. Arch. Intern. Med. 161: 1413–1419. 11386890 10.1001/archinte.161.11.1413

[53] Hartz I., Sakshaug S., Furu K., Engeland A., Eggen A. E., Njølstad I., and Skurtveit S. 2007 Aspects of statin prescribing in Norwegian counties with high, average and low statin consumption—an individual-level prescription database study. BMC Clin. Pharmacol. 7: 14. 18053228 10.1186/1472-6904-7-14 PMC2234392

[54] Barrett E. S., Parlett L. E., Windham G. C., and Swan S. H. 2014 Differences in ovarian hormones in relation to parity and time since last birth. Fertil. Steril. 101: 1773–1780.e1. 24684956 10.1016/j.fertnstert.2014.02.047 PMC4041832

[55] Toriola A. T., Vääräsmäki M., Lehtinen M., Zeleniuch-Jacquotte A., Lundin E., Rodgers K-G., Lakso H-A., Chen T., Schock H., Hallmans G., 2011 Determinants of maternal sex steroids during the first half of pregnancy. Obstet. Gynecol. 118: 1029–1036. 22015870 10.1097/AOG.0b013e3182342b7f PMC3207141

[56] Arslan A. A., Zeleniuch-Jacquotte A., Lukanova A., Afanasyeva Y., Katz J., Levitz M., Del Priore G., and Toniolo P. 2006 Effects of parity on pregnancy hormonal profiles across ethnic groups with a diverse incidence of breast cancer. Cancer Epidemiol. Biomark. Prev. 15: 2123–2130. 10.1158/1055-9965.EPI-06-0470 17119037

[57] Windham G. C., Elkin E., Fenster L., Waller K., Anderson M., Mitchell P. R., Lasley B., and Swan S. H. 2002 Ovarian hormones in premenopausal women: variation by demographic, reproductive and menstrual cycle characteristics. Epidemiology. 13: 675–684. 12410009 10.1097/00001648-200211000-00012

[58] Dorgan J. F., Reichman M. E., Judd J. T., Brown C., Longcope C., Schatzkin A., Campbell W. S., Franz C., Kahle L., and Taylor P. R. 1995 Relationships of age and reproductive characteristics with plasma estrogens and androgens in premenopausal women. Cancer Epidemiol. Biomark. Prev. 4: 381–386. 7655334

[59] Bernstein L., Pike M. C., Ross R. K., Judd H. L., Brown J. B., and Henderson B. E. 1985 Estrogen and sex hormone-binding globulin levels in nulliparous and parous women. J. Natl. Cancer Inst. 74: 741–745. 3857369

[60] Gunderson E. P., Whitmer R. A., Lewis C. E., Quesenberry C. P., West D. S., and Sidney S. 2005 Do long-term HDL-C declines associated with a first birth vary by apo E phenotype? The Coronary Artery Risk Development in Young Adults (CARDIA) study. J. Womens Health (Larchmt.). 14: 917–928. 16372893 10.1089/jwh.2005.14.917 PMC3146172

[61] Zeljkovic A., Vekic J., Spasic S., Jelic-Ivanovic Z., Spasojevic-Kalimanovska V., Gojkovic T., Ardalic D., Mandic-Markovic V., Cerovic N., and Mikovic Z. 2013 Changes in LDL and HDL subclasses in normal pregnancy and associations with birth weight, birth length and head circumference. Matern. Child Health J. 17: 556–565. 22527773 10.1007/s10995-012-1031-x

[62] Gordon T., Castelli W. P., Hjortland M. C., Kannel W. B., and Dawber T. R. 1977 High density lipoprotein as a protective factor against coronary heart disease. The Framingham Study. Am. J. Med. 62: 707–714. 193398 10.1016/0002-9343(77)90874-9

[63] Kannel W. B. 1983 High-density lipoproteins: epidemiologic profile and risks of coronary artery disease. Am. J. Cardiol. 52: 9B–12B. 10.1016/0002-9149(83)90649-5 6577783

[64] Fisher E. A., Feig J. E., Hewing B., Hazen S. L., and Smith J. D. 2012 HDL function, dysfunction, and reverse cholesterol transport. Arterioscler. Thromb. Vasc. Biol. 32: 2813–2820. 23152494 10.1161/ATVBAHA.112.300133 PMC3501261

[65] Khera A. V., Cuchel M., de la Llera-Moya M., Rodrigues A., Burke M. F., Jafri K., French B. C., Phillips J. A., Mucksavage M. L., Wilensky R. L., 2011 Cholesterol efflux capacity, high-density lipoprotein function, and atherosclerosis. N. Engl. J. Med. 364: 127–135. 21226578 10.1056/NEJMoa1001689 PMC3030449

