# Supplementary material for: Does pregnancy alter life-course lipid trajectories? Evidence from the HUNT Study in Norway
Source: J Lipid Res. 2018 Oct 12;59(12):2403–12. doi: 10.1194/jlr.P085720 (PMC6277164; doi:10.1194/jlr.P085720)
Supplement: Supplemental Data [file supp_59_12_2403__index.html]

Does Pregnancy Alter Life Course Lipid Trajectories? Evidence from the HUNT Study in Norway — Does pregnancy alter life-course lipid trajectories? Evidence from the HUNT Study in Norway — Supplemental Data 

# Does pregnancy alter life-course lipid trajectories? Evidence from the HUNT Study in Norway

## Supplemental Data

- Supplemental Data (.docx, 865 KB) - Supplemental Methods Sections, Tables, and Figures for manuscript
