# Supplementary material for: Does pregnancy alter life-course lipid trajectories? Evidence from the HUNT Study in Norway
Source: J Lipid Res. 2018 Oct 12;59(12):2403–12. doi: 10.1194/jlr.P085720 (PMC6277164; doi:10.1194/jlr.P085720)

SUPPLEMENTAL DATA

**Supplemental Methods.** Additional statistical analysis details

Section 1: Analyses comparing parous and nulliparous women

The basic form of the linear mixed effects model used in analyses comparing parous and nulliparous women (Figure 2 and Table 2) is shown below without inclusion of adjustment covariates for simplicity:

$y_{ij}=\beta_{0}+\beta_{1}P_{i}+\beta_{2}I_{ij}+\beta_{3}T_{ij}+\beta_{4}A_{ij}+\beta_{5}A_{ij}P_{ij}+\sum_{k=1}^{n-2} \left( \beta_{6.k}+\beta_{7.k}P_{i} \right)S_{ijk}+$

$u_{0i}+u_{1i}A_{ij}+e_{ij}$ **(Equation 1)**

where $y_{ij}$ are lipid levels for woman $i$ at time $j$

$P_{i}$ indicates the final parity status for woman $i$ (i.e. none vs one or more births)

$I_{ij}$ indicates whether the measurement preceded or followed first birth

$T_{ij}$ is continuous time in years since first birth

$A_{ij}$ is the woman’s age in years at the time of measurement

$S_{ijk}$ are splines for age with $n$ knots and are calculated as follows:

$$S_{ijk}=\frac{{(A_{ij}-t_{k})}_{+}^{3}-(t_{n}-t_{n-1})^{-1}\{(A_{ij}-t_{n-1})_{+}^{3}\left( t_{n}-t_{k} \right)-(A_{ij}-t_{n})_{+}^{3}\left( t_{n-1}-t_{k} \right)\}}{(t_{n}-t_{1})^{2}}$$

where $t_{k}$,$k=1,\ldots, n$ are the knot values and

${(x)}_{+}=\left\{ \begin{aligned} x, if x>0 \\ 0, if x\leq0 \end{aligned} \right.$

and $u_{0i}$ and $u_{1i}$ are a random intercept and slope for woman $i$

This model was the best fit after comparing the Bayesian Information Criterion for models with age modeled as linear or with linear splines and among different choices for the number of knot points. These models adjusted for baseline covariates using main effect terms as well as interactions with the pre- vs post-first birth indicator ($I_{ij}$), time since first birth ($T_{ij})$, and age splines ($A_{ij}$ and $S_{ijk}$).

Section 2: Analyses of only parous women

The basic form of the linear mixed effects model used in analyses of only parous women (Table 3) is shown below also without inclusion of adjustment covariates for simplicity:

$y_{ij}=\beta_{0}+\beta_{1}I_{ij}+\beta_{2}T_{ij}+\beta_{3}A_{ij}+\sum_{k=1}^{n-2} (\beta_{4.k}S_{ijk})+u_{0i}+u_{1i}A_{ij}+e_{ij}$ **(Equation 2)**

Models used in Table 3 included main effect terms for baseline covariates and, in Table 3 Model 2, time-varying covariates. We also included interaction terms between these covariates and pre- vs post-first birth indicator ($I_{ij}$) and between BMI and the age splines ($A_{ij}$ and $S_{ijk}$). Including additional interaction terms in these models led to difficulties with convergence for the imputation models; however, as a diagnostic test, we ran a complete case analysis including all possible interaction terms between covariates and the continuous terms for time since pregnancy as well as between covariates and age splines and did not find a meaningful change in effect estimates of interest. Analyses of multiple births (Table 4) used the same model form but with separate terms for the effect of pregnancy ($I_{ij}$ and $T_{ij}$) for each birth.

**
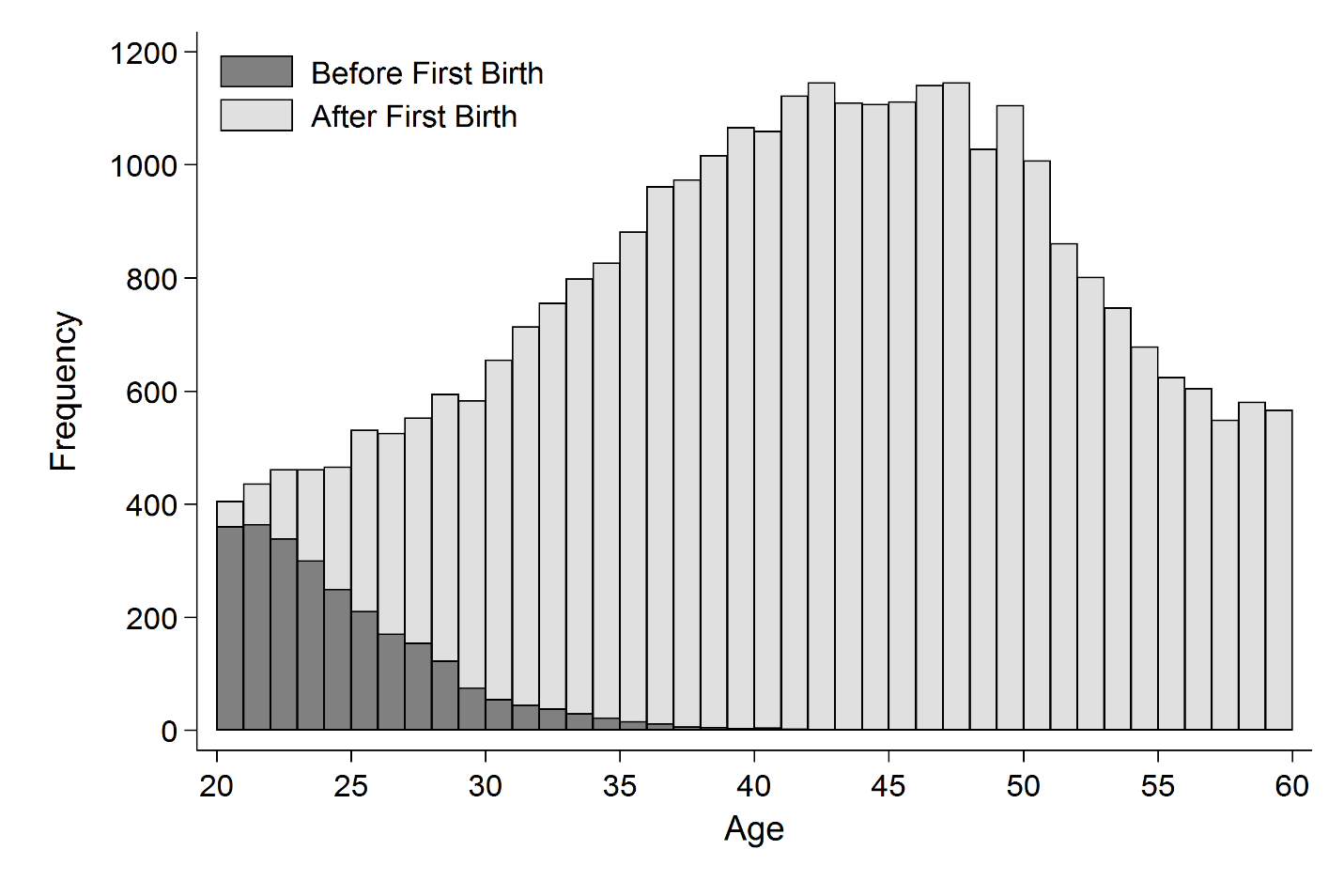
**

**Supplemental Figure S1.** Timing of lipid measurements by age and with respect to first birth, parous HUNT2 and HUNT3 study participants (n=22,047 women)

**Supplemental Table S1.** Technical details for lipid measurements

|  | **Measurement Method** | |
| --- | --- | --- |
|  | **HUNT2** | **HUNT3** |
| All Lipids | Measured at Levanger Hospital’s Central Laboratory using an Hitachi 911 Autoanalyzer (Mito, Japan) with reagents from Boehringer Mannheim (Mannheim, Germany) | Measured at Levanger Hospital’s Central Laboratory using an Architect ci8200 with reagents from Abbott (Abbott Ireland, Longford, Ireland; and Abbott Laboratories, Abbott Park, IL) |
| Total Cholesterol | enzymatic cholesterol esterase methods | enzymatic cholesterol esterase methods |
| HDL-C | enzymatic cholesterol esterase methods | accelerator selective detergent methods |
| Triglycerides | enzymatic colorimetric method | glycerol phosphate oxidase methods |


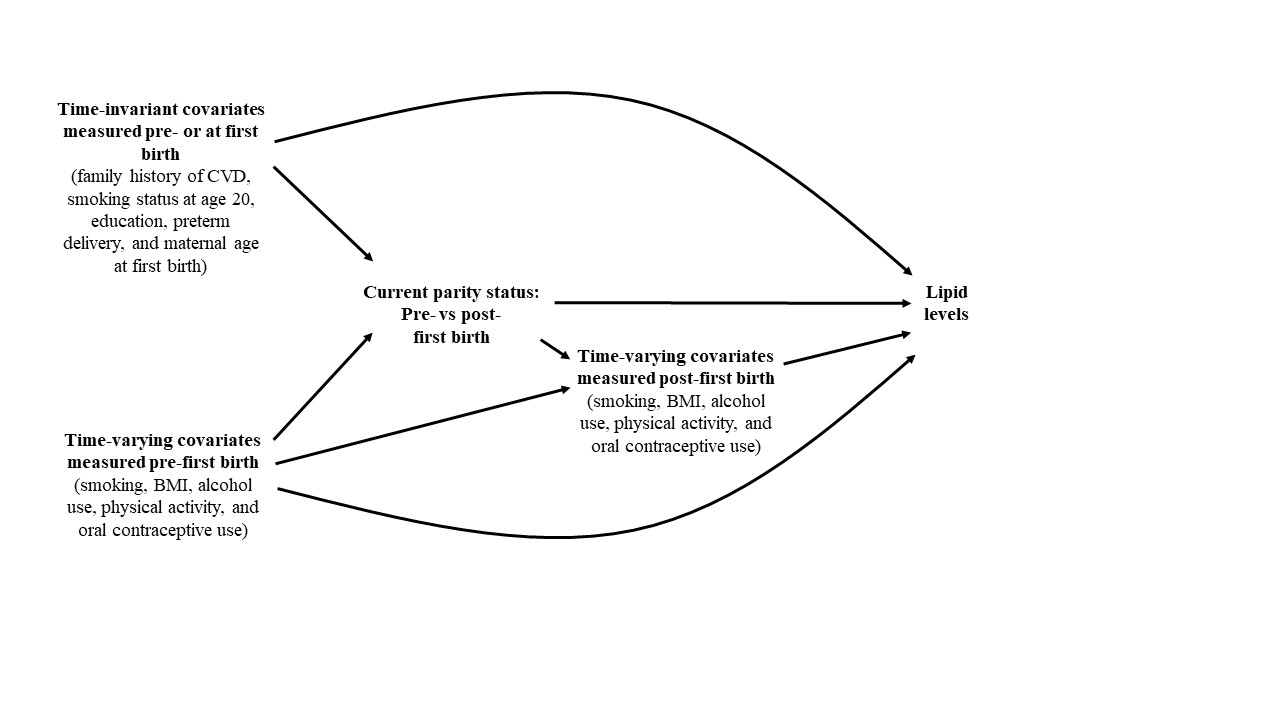


**Supplemental Figure S2.** Study causal diagram. Primary analyses control for time-invariant covariates measured pre-first birth and secondary analyses include all covariates shown. In analyses of breastfeeding length, we considered it a category of the exposure (pre-first birth, post-first birth and did not breastfeed, post-first birth and breastfed <3 months, post-first birth and breastfed 3-6 months, and post-first birth and breastfed >6 months); however, specific definitions of effects cannot be easily shown on causal diagrams. Nulliparous women were used in analyses to represent background age and secular trends independent of parity.

CVD=cardiovascular disease; BMI=body mass index


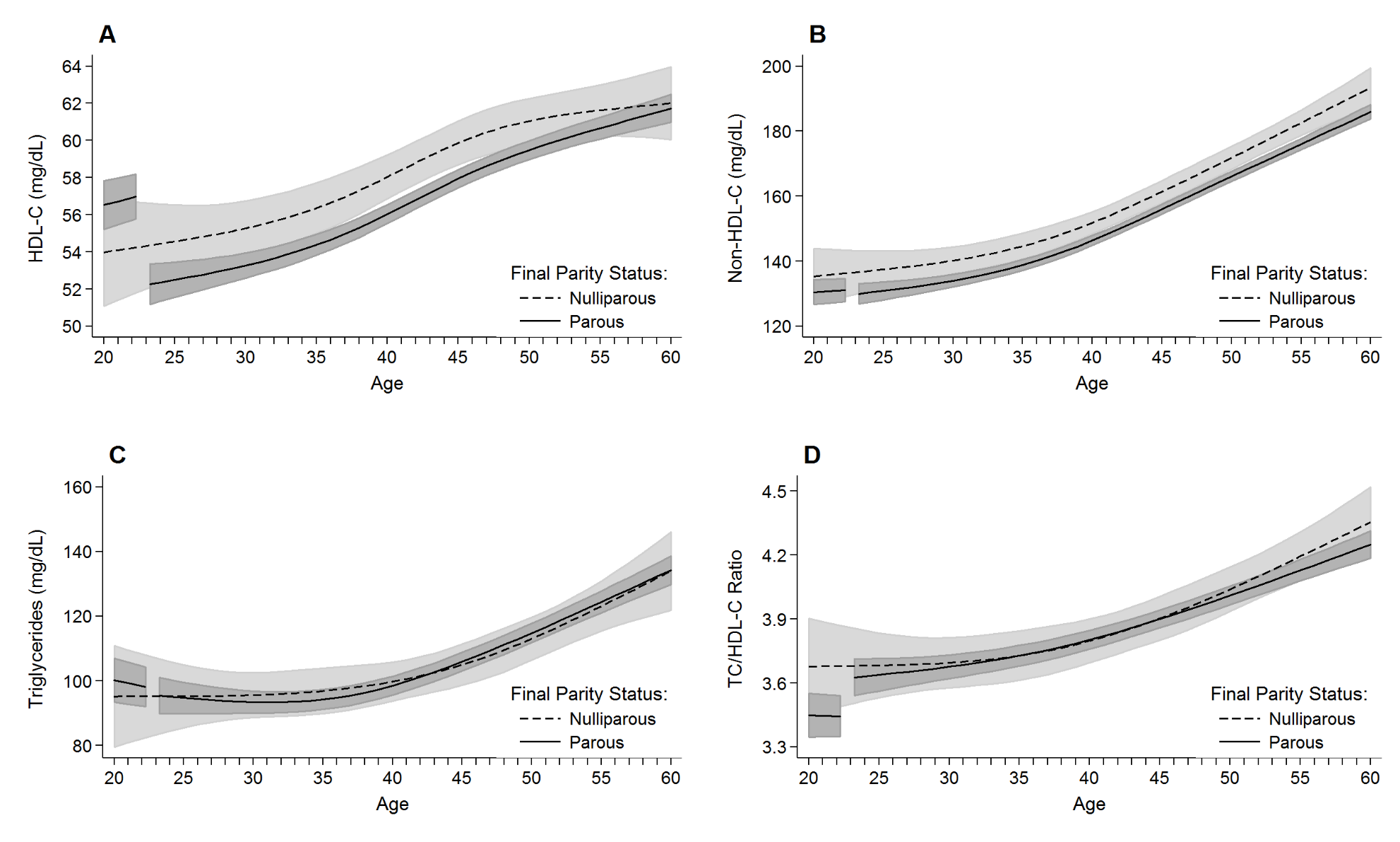
 **Supplemental Figure S3.** Predicted lipid trajectories and 95% confidence intervals based on final parity status for women with two measurements (n=11,983) of A) high-density lipoprotein cholesterol (HDL-C), B) non-HDL-C, C) triglycerides, and D) total cholesterol (TC) to HDL-C ratio. Estimates for parous women are predicted for hypothetical women with first birth at age 23. Gaps represent pregnancy and 3 months postpartum. Predictions are at the mean level for covariates: HUNT survey, time since last meal, education, smoking initiation by age 20, and family history of cardiovascular disease.

**Supplemental Table S2.** Mean within-woman change in lipids between HUNT2 and HUNT3 (n=1,035)^a^

| **No. of births between HUNT2 and HUNT3** |  | **HDL-C (mg/dL)** | | | **Non-HDL-C (mg/dL)** | | | **Triglycerides (mg/dL)** | | | **TC/HDL-C Ratio** | | |
| --- | --- | --- | --- | --- | --- | --- | --- | --- | --- | --- | --- | --- | --- |
|  | N | Change | 95% CI | P value | Change | 95% CI | P value | Change | 95% CI | P value | Change | 95% CI | P value |
| None | 385 | (ref) |  |  | (ref) |  |  | (ref) |  |  | (ref) |  |  |
| Any | 650 | -5.2 | -7.0, -3.4 | <0.001 | -3.8 | -8.2, 0.7 | 0.10 | -6.7 | -15.7, 2.3 | 0.15 | 0.19 | 0.06, 0.31 | 0.003 |
| 1 | 147 | -3.8 | -6.2, -1.4 | 0.002 | -0.4 | -6.5, 5.7 | 0.90 | -7.9 | -20.2, 4.4 | 0.21 | 0.24 | 0.07, 0.40 | 0.005 |
| 2 | 336 | -5.1 | -7.1, -3.2 | <0.001 | -5.3 | -10.3, -0.3 | 0.04 | -4.4 | -14.4, 5.7 | 0.39 | 0.13 | -0.00, 0.27 | 0.05 |
| ≥ 3 | 167 | -7.1 | -9.5, -4.7 | <0.001 | -4.0 | -10.1, 2.1 | 0.20 | -10.8 | -23.0, 1.5 | 0.09 | 0.25 | 0.08, 0.41 | 0.004 |

HDL-C=high density lipoprotein cholesterol; TC=total cholesterol

^a^Estimates are adjusted for age, education, and family history of cardiovascular disease at baseline (HUNT2) and change in smoking status, body mass index, and time since last meal from HUNT2 to HUNT3.

**Supplemental Figure S4.** Predicted lipid trajectories and 95% confidence intervals based on final parity status, adjusted for menopause and hormone replacement therapy, including A) high-density lipoprotein cholesterol (HDL-C), B) non-HDL-C, C) triglycerides, and D) total cholesterol (TC) to HDL-C ratio. Estimates for parous women are predicted for women with a hypothetical first birth at age 23. Gaps represent pregnancy and 3 months postpartum. Predictions are at the mean level for covariates: HUNT survey, time since last meal, education, smoking initiation by age 20, family history of cardiovascular disease, menopause transition, and hormone replacement therapy use.


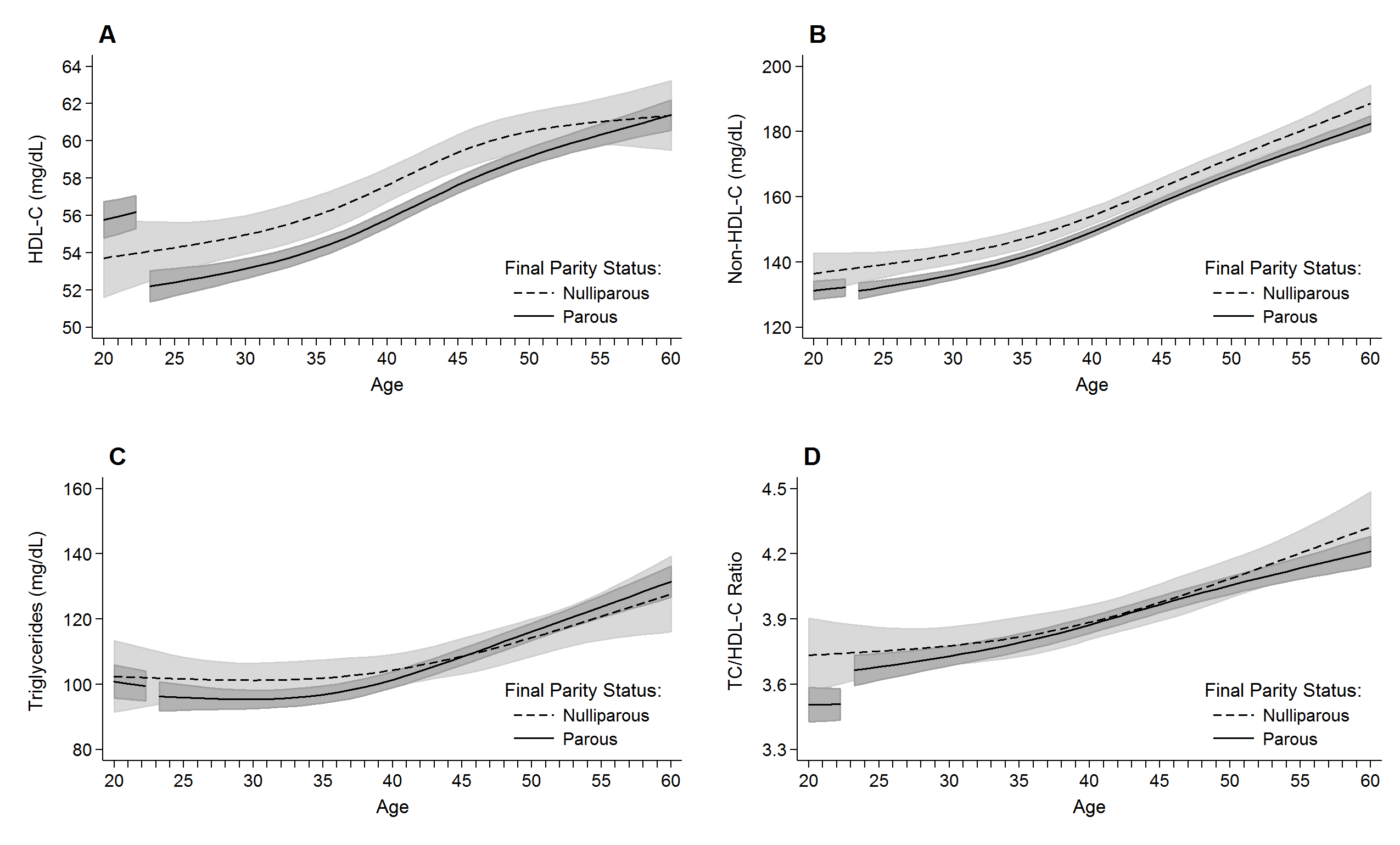

Supplement: Supplemental Data [file 10.1194_P085720_jlr.P085720-1.docx]
